# GDF-5 promotes epidermal stem cells proliferation via Foxg1-cyclin D1 signaling

**DOI:** 10.1186/s13287-020-02106-7

**Published:** 2021-01-07

**Authors:** Xiaohong Zhao, Ruyu Bian, Fan Wang, Ying Wang, Xue Li, Yicheng Guo, Xiaorong Zhang, Gaoxing Luo, Rixing Zhan

**Affiliations:** 1 Institute of Burn Research; State Key Laboratory of Trauma, Burn and Combined Injury; Southwest Hospital, The Third Military Medical University (Army Medical University), Chongqing, 400038 China; 2 Department of Plastic and Reconstructive Surgery, Southwest Hospital, The Third Military Medical University (Army Medical University), Chongqing, 400038 China

**Keywords:** GDF-5, Mouse epidermal stem cells, Foxg1, Cell proliferation, Cyclin D1

## Abstract

**Objective:**

Epidermal stem cells (EpSCs) can self-renew, which are responsible for the long-term maintenance of the skin, and it also plays a critical role in wound re-epithelization, but the mechanism underlying EpSCs proliferation is unclear. GDF-5, also known as BMP-14, is a member of the BMP family and can be used as a self-renewal supporter. Here, we studied the effects of GDF-5 on mouse EpSCs proliferation mechanism in wound healing.

**Methods:**

Firstly, the effects of GDF-5 on EpSCs proliferation was tested by using CCK8 reagent and PCNA expression was analyzed by Western blotting. Secondly, we screened genes that promote EpSCs proliferation in the FOX and cyclin family by qPCR, and then the protein expression level of the selected genes was further analyzed by Western blotting. Thirdly, siRNA plasmids and pAdEasy adenovirus were transfected or infected, respectively, into mouse EpSCs to detect the effect of target genes on GDF-5-induced cell proliferation. Furthermore, we injected GDF-5 to a deep partial thickness burn mouse model for finding out whether EpSCs proliferation can be detected by immunohistochemical. Finally, the relevant target genes were analyzed by qPCR, immunoblotting, and dual-luciferase reporter gene detection.

**Results:**

We discovered that 100 ng/ml recombinant mouse GDF-5 was the optimal concentration for promoting mouse EpSCs proliferation. Through preliminary screened by qPCR, we found that Foxg1 and cyclin D1 could be the downstream molecules of GDF-5, and the results were confirmed by Western blotting. And the effect of GDF-5 on mouse EpSCs proliferation was adjusted by Foxg1/cyclin D1 in vitro and in vivo. Besides, GDF-5-induced transcription of cyclin D1 was regulated by Foxg1-mediated cyclin D1 promoter activity.

**Conclusion:**

This paper showed that GDF-5 promotes mouse EpSCs proliferation via Foxg1-cyclin D1 signal pathway. It is suggested that GDF-5 may be a new approach to make EpSCs proliferation which can be used in wound healing.

## Introduction

The epidermis is derived from ectoderm cells during embryonic development, these cells go through a layering process to form basal, spinous, and granular layers [1]. The skin epidermis contains different appendages including sweat glands, hair follicles, and sebaceous glands, which is responsible for immune regulation, pigmentation, and sensory function [2]. The self-renewal and damage repairing of skin tissue mainly depend on the compensatory proliferation and differentiation of EpSCs [3]. After Billingham and Reynolds firstly reported skin cell transplantation for wound healing in 1952 [4], EpSCs have been used in clinical practice, repairing of burns, acute trauma, and skin damage caused by certain diseases [5–7]. But the expansion of EpSCs always is a choke point in its clinical application. The physiological state of EpSCs can be affected by different signaling pathways, including MAPK (mitogen-activated protein kinase) [8, 9], Wnt (wingless) [10, 11], and TGF-β (transforming growth factor-beta) [12] signaling. BMP (bone morphogenetic protein) belongs to the TGF-β family and can stimulate cell proliferation [13].

Extracellular BMP binds with cell membrane receptors to initiate downstream signaling pathways, and the signal molecule is translocated to the nucleus, where it is combined with a nuclear transcription factor to regulate gene expression [12]. According to the report, BMP-4 supports self-renewal by inhibiting MAPK pathways in mouse embryonic stem cells [14]. Growth/differentiation factor 5 (GDF-5) is a BMP family member [15], also known as CDMP-1 and BMP-14. Studies suggest that GDF-5 affects angiogenesis [16], migration [16], apoptosis [17], and differentiation [18] in vitro. Syed H. E and his colleagues also discovered that GDF5-induced p38-MAPK signaling in fibroblasts regulates cardiac repairing after myocardial infarction [15]. The predecessors conducted a preliminary study on the effect of GDF-5 in wound repairing [13], so we speculate that the increase of GDF-5 may promote the proliferation of EpSCs, but the specific proliferation mechanism of EpSCs promoted by GDF-5 has not been reported. Recent studies have found that FOX (Fork head box) and cyclin are involved in the promotion of cell proliferation by BMP [19].

FOX is a kind of nuclear transcription factors family. The activity of FOX protein can be regulated by phosphorylation, acetylation, and protease hydrolysis [20]. It is known that PI3K-AKT/PKB (phosphoinositide-3-kinase–protein kinase B/Akt), TGFβ-Smad and MAPK signaling pathways can affect the level of FOX family proteins [19]. Foxa1, Foxc1, Foxd3, Foxo3, Foxg1, Foxp1, and Foxm1 are associated with cell proliferation [21]. The Foxg1 gene is a dose-sensitive gene, and it can antagonize the pro-apoptotic effect of Foxo3 [22] and promote hepatocellular carcinoma epithelial-mesenchymal transition [23]. As Shasha Zhang’s reported, knocking out the Foxg1 gene will increase differentiation of newborn mouse cells [24]. Studies have found that cyclin D plays an important role in cell proliferation, which has three subfamilies: cyclin D1, cyclin D2, and cyclin D3. It mainly initiates signal cascade after binding and activating CDK (4 or 6) (cyclin-dependent kinase 4 or 6) which promoting cell proliferation [25, 26]. Julie A. Siegenthaler reported that Foxg1 was associated with cyclin in promoting intermediate progenitor cell proliferation [27]. Besides, the Wnt/cyclin D1 pathway has a dedifferentiating effect for differentiated epidermal cells [28]. In our previous research, we found that cyclin D1 is an important downstream signaling molecule in the proliferation of EpSCs [29].

In this paper, we conducted an integral study in vitro and in vivo conditions and carried out necessary tests. We sought to elucidate the effects of GDF-5 on mouse EpSCs proliferation mechanisms in wound healing. We also hope to find new wound repair targets through our research and provide new strategies for clinical research.

## Methods and reagents

### Animals

Both male and female C57BL/6 mice were used in this study. They were obtained from the Experimental Animal Department of the Army Military Medical University, China. All animal procedures were approved by the Committee on the ethics of Animal Experiments of the Third Military Medical University and were conducted in accordance with the guidelines of the Experimental Animal Department of the Army Military Medical University. The animals were individually housed in plastic cages under standard conditions (temperature, 25 °C; relative humidity, 50%; and circadian rhythm, 12 h). Animals were provided cold boiled water and rodent food, and allow them to acclimate to the facility for 1 week before the experiment. All surgeries were performed under 0.1% sodium pentobarbital anesthesia, and all efforts were made to minimize suffering. The wound needs to be covered with sterile oil gauze to prevent infection.

### Preparation of mouse primary EpSCs

The preparation of primary EpSCs from newborn mice (0–2 days) was described in our previous studies [30]. Firstly, the neonatal mice were euthanized by cervical dislocation. Secondly, soaked for 1 min in 75% ethanol and washed twice with sterile PBS. Thirdly, the back skin was separated with sterile surgical instruments and incubated with 0.5% dispase II (Gibco, #17105041) overnight at 4 °C. Next, the skin was washed three times with sterile PBS and separated carefully and the epidermis was dissociated with 0.25% Trypsin (Gibco, #25200056) at 37 °C for 10 min; the single-cell suspension was passed through a 70-μm filter (BD Falcon #352350) into a sterile 15-ml tube. Then, the cell suspension was centrifuged at 1000 rpm for 5 min, removed supernatant, and resuspended cells in K-SFM (Gibco, 10744019) supplemented with 0.2 ng/ml recombinant mouse EGF (stem cell, #78016), 100 ng/ml Cholera toxin, 30 mg/ml BPE (bovine pituitary extract), 0.05 mM calcium chloride, and 100 U/ml of streptomycin and penicillin. Follow 2.5 × 10^6 cells/T25 to quickly adhere to the bottom for 10 min, change the medium. Finally, the cells were cultured and the medium was changed every 2–3 days.

### Flow cytometry analysis

When second-generation (P2) cells confluence become ~ 70% after TrypLE™ select (Gibco, #12563029) passaged. Flow cytometry analysis of the purity of passaged EpSCs: Collect EpSCs at a density of 10^6 cell/ml, and then add antibodies, Santa SC23372-CD71-PE 5 μl/EP tube, Santa SC19622-CD49f-FITC 5 μl/EPT tube, test after 60 min incubation at 4 °C. Finally, resuspended in 0.5 ml of PBS, and then subjected to flow cytometry analysis.

### Cell proliferation assay

Possible proliferation was assessed by cell viability using Cell Counting Kit-8 (CCK-8) (Beyotime, C0038, China) according to the manufacturer’s instructions. When first-generation (P1) cells confluence become ~ 70%, collect EpSCs at a density of 2 × 10^5 cell/ml. Then, the 2000 EpSCs were seeded in 96-well plates (100 μl/well) and treated with 0, 1, 50, 100, 500, and 1000 ng/ml of GDF-5 (Beyotime, P6193, China) for 12, 24, 48, and 72 h. After that, 10 μl CCK-8 solution was added to 96-well and incubated for 2–4 h at 37 °C. Absorbance was measured at 450 nm with a microplate reader (Spectra Max 190; Molecular Devices).

### Adenovirus infection and siRNA transient transfection

Adenovirus transfection and siRNA interference protocol were as previously described [31, 32]. Adenovirus transfection was made when EpSCs reached 70% confluence and then aspirated the medium and added fresh medium. After that, added 10 μl Myc adenovirus and 10 μl Foxg1 adenovirus and 10 μl empty vector to each group. After 24 h’ transfection, we observed the fluorescence intensity and expression ratio with a fluorescence microscope and changed the medium. After 48 h’ transfection, cells were collected for subsequent experiments. Specific cyclin D1 siRNA and control siRNA were purchased from Thermo Fisher Scientific. EpSCs preparation method is as described above. Mouse EpSCs were transfected with siRNA according to the manufacturer’s instructions. The efficiency of siRNA interference was analyzed by the following Western blotting.

### Western blotting (WB)

The levels of C Foxg1, cyclin D1, PCNA, and GAPDH protein were detected by WB. GDF-5 was treatment EpSCs for 24 h, and then collect cells. EpSCs protein samples were prepared using RIPA lysis buffer (Beyotime, P0013B, China), which contained protease and phosphatase inhibitors. It was quantified using BCA protein evaluation kit (Beyotime, P0012S, China). Next, 30 μg/each sample of protein was loaded onto 10% SDS-PAGE and transferred to PVDF membrane (Beyotime, FFP24, China). Then, the membrane was blocked with a 5% milk solution (w/v) at room temperature for 2 h. Then, the primary antibody was incubated overnight at 4 °C. The primary antibody was diluted according to the following ratio: PCNA (ab92552, 1:5000), Foxg1 (ab196868, 1:5000), cyclin D1 (ab16663, 1:5000), and GAPDH (ab181606, 1:10,000). All antibodies were purchased from Abcam (Cambridge, Massachusetts, USA). After incubating the primary antibody, the membrane was washed three times and incubated with goat anti-rabbit IgG (H + L) (1:10,000) (Abcam, ab6702) for 1 h. The bands were visualized by using the BeyoECL Plus (Beyotime, P0018M, China), and the bands were detected using Image Quant LAS 4000 s (GE, USA) [33].

### Real-time quantitative PCR (qPCR)

GDF-5 treatment EpSCs for 24 h, and then collect cells. We used RNAiso Plus (Takara, # 9109) to extract RNA following the instructions, and measured the A260 and A280 values of the sample. Next, qPCR was performed. The first step is to remove the genomic DNA (42 °C, 2 min; 4 °C hold), the second step is the reverse transcription reaction (37 °C, 15 min; 85 °C, 5 s; 4 °C hold), and finally the real-time PCR reaction (95 °C, 30 s; go to 39 (40 cycles), 95 °C, 5 s, 60 °C, 30 s; melt curve). The primescript RT reagent kit with gDNA Eraser (Takara, # RR047A) and TB green Premix Ex Taq (Takara, # RR820A) were used. Real-time PCR analysis of mouse cDNA was performed using the 7500 qPCR System (Applied Biosystems). GAPDH serves as an internal reference. Primers synthesized by Sangon Biotech (Shanghai), and primer sequences are listed in Table 1.

**Table 1:** Primers for the RT-qPCR

Primer name	Sequence (5′ to 3′)	Length	Tm (°C)
GAPDH-F	GGTTGTCTCCTGCGACTTCA	20	57.5
GAPDH-R	TGGTCCAGGGTTTCTTACTCC	21	56.5
Foxa1-F	TTACAAGGATGCCTCTCCA	19	52.6
Foxa1-R	TGGCTCTCTGAAAAGCAAG	19	52.4
Foxc1-F	GGATCGGCTTGAACAACT	18	52.4
Foxc1-R	AGAGTGCCGGGAATAGG	17	54.0
Foxd3-F	CGTAGAGAAGCGTCGAGGA	19	56.6
Foxd3-R	GGCAAAGGAGGTGTGAGTG	19	56.5
Foxg1-F	AACGGGCTGAGTGTGGA	17	58.8
Foxg1-R	CAGGGGTTGAGGGAGTAGG	19	57.6
Foxo3-F	GAGGATTCGGCCATGCT	17	55.4
Foxo3-R	TTCCTTGGTTGCCCAGAG	18	55.1
Foxp1-F	TGCGCTGGACGATAGAA	17	53.4
Foxp1-R	ATGCAGGTGGGTCATCA	17	53.5
Cyclin C-F	ATGCTTGGTAATTGATTTGCT	21	49.5
Cyclin C-R	CAGGGGTTGAGGGAGTAGG	19	57.6
Cyclin D1-F	ACCCTGACACCAATCTCCT	19	55.6
Cyclin D1-R	CTCCTTCTGCACGCACTT	18	55.6
Cyclin D2-F	CCGTTCTTGGCTCTGGT	17	55.1
Cyclin D2-R	AGGCACCTGTTGAAACTGA	19	53.9
Cyclin D3-F	AAACCACGCCCCTGACT	17	57.2
Cyclin D3-R	AGGTCCCACTTGAGCTTCC	19	57.4
Cyclin E-F	CCCAAGTCCTGAGCCAT	17	54.7
Cyclin E-R	TCGGAGCCACCTTCTTC	17	54.5

### Dual-luciferase assay

Follow our previous method [32]. Firstly, construction of reporter gene plasmid. The mouse cyclin D1 promoter sequence was cloned into pGL3-basic plasmid vector to obtain pF1, pF2, pF3, pF4, pF5, and pF6 plasmid. Mutated pF2 was generated by using the Mutant Best Kit (Takara, China). Secondly, transfect cells. At 48 h after pAdEasy-Foxg1 or pAdEasy-Myc transfection, the cells were transfected with the above luciferase reporter expression vectors using Lipofectamine 2000 for the promoter assay, respectively. And then, we used a multi-functional microplate reader to detect the expression level of the reporter gene according to the manufacturer’s instructions (Promega, E2940).

### EpSCs proliferation assay in vivo

A deep partial-thickness burn mouse model was made as follows description [34], and EpSCs were labeled with BrdU in mouse skin. Neonatal C57BL/6 mice were intraperitoneally injected with BrdU (50 mg/kg body weight, Sigma) twice daily for 3 days, beginning on day 3 after birth. Skin cells retaining BrdU were identified as EpSCs after 7 weeks. Next, an anesthetic was injected intraperitoneally. Each gram of body weight was injected with 0.1% sodium pentobarbital at a dose of 10 μl; a metal plate (Shandong Academy of Medical Science, China) with a diameter of 1.5 cm and weight of 0.5 kg was used to induce deep partial-thickness burns. The metal plate was heated to 70 °C and was placed evenly on the shaved mouse dorsum for 3 s to form a scald model. The skin wounds were covered with sterile oil gauze to prevent infection; mice were individually housed in plastic cages under standard conditions.

The 36 mice were divided randomly into 6 groups: control (normal saline), pAdEasy-Myc, cyclin D1 siRNA, GDF-5, pAdEasy-Foxg1 + GDF-5, and cyclin D1 siRNA + GDF-5. After modeling, control group (normal saline 0.05 ml/g body weight, i.p.), pAdEasy-Myc and pAdEasy-Foxg1 and cyclin D1 siRNA (0.5 ml of PBS containing 2.5 × 10^8^ PFU virus, wound margin five points, s.c.), GDF-5 (0.05 ml/g body weight, i.p.), and GDF-5’s concentration is 10 μg/ml in normal saline. Mice were sacrificed after 24 h and using immunofluorescence assay BrdU and PCNA.

### Tissue immunofluorescence analysis

An anesthetic was injected intraperitoneally. Each gram of body weight was injected with 0.1% sodium pentobarbital at a dose of 10 μl, and the mouse was euthanized by cervical dislocation. The wound was biopsy, fixed in 4% paraformaldehyde, and cut into 4 μm slices. Next, antigen retrieval was performed using citrate buffer in a pressure cooker at 95 °C for 30 min and each group was blocked in 10% goat serum (16210064; Gibco) for 30 min at 37 °C. Then, antibody incubation PCNA (ab92552,1:1000) at 4 °C overnight. And then drop the corresponding fluorescent secondary antibody (Goat Anti-Rabbit IgG H&L (Cy5®) (ab6564)), DAPI counterstain. Finally, dehydration and fix the mount, confocal laser observation.

### Statistical methods

All data were presented as the mean ± standard deviation (SD) with at least three independent experiments and analyzed using GraphPad Prism 7.0 software. Statistical significance was evaluated by one-way ANOVA or *t* test. *P* < 0.05 was considered statistically significant.

## Results

### Effect of GDF-5 on mouse EpSCs proliferation

**Fig. 1:**
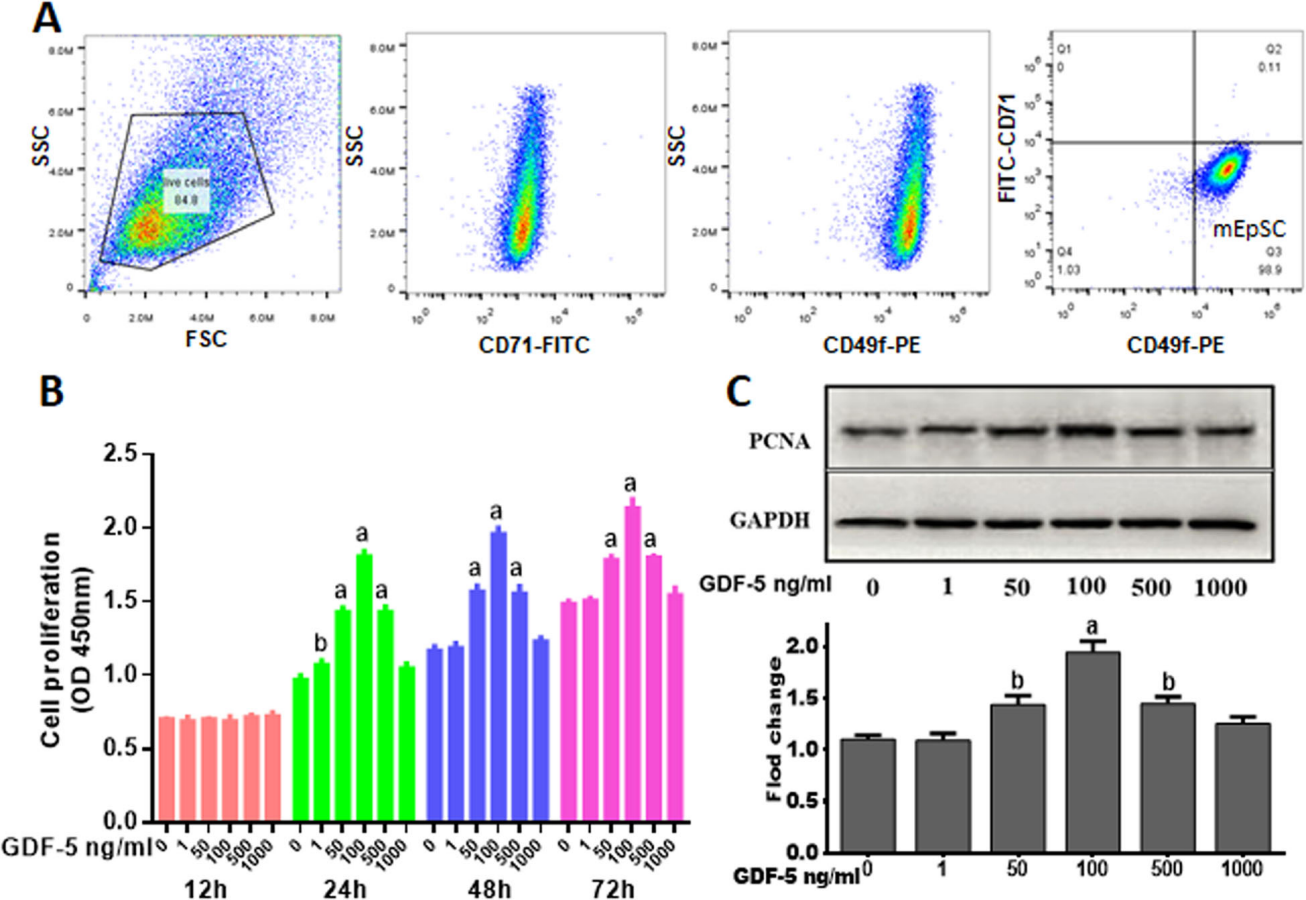
Effect of GDF-5 on mouse EpSCs proliferation in vitro. **a** Flow cytometry was used to analyze the passaged EpSCs (P2). After the cells were passaged twice, the cells were labeled with CD49f and CD71 antibodies. EpSCs expressed high levels of CD49f and low levels of CD71. **b** Mouse primary EpSCs were treated with 0, 1, 50, 100, 500, and 1000 ng/ml of GDF-5 for 12, 24, 48, and 72 h. Cell proliferation was measured by CCK8. **c** EpSCs were treated by GDF-5 for 24 h, and the PCNA levels were analyzed by WB. The data were shown as the means ± SD of three independent experiments. ^a^*P* < 0.01 vs. control (0 ng/ml GDF-5 as control), ^b^*P* < 0.05 vs. control

Mouse EpSCs were defined as our previously described [31, 35]. The purity of EpSCs was analyzed by flow cytometry, as shown in Fig. 1a, and the proportion of EpSCs is about 99%. To illustrate the effect of GDF-5 on mouse EpSCs proliferation, we used CCK-8 assay the proliferation effect of GDF-5 at different time points, and the results showed that 24 h has the best effect on promoting cell proliferation (0.84-fold) when GDF-5 was 100 ng/ml (Fig. 1b), so 24 h was used in the following study. Because the PCNA is a marker that reflects the state of cell proliferation [36], our further analysis of GDF-5 promoting EpSCs proliferation at 24 h found that the proliferation-associated PCNA protein was significant at 100 ng/ml (*P* < 0.01) in response to GDF-5 (Fig. 1c).

### The possible downstream molecules of GDF-5

**Fig. 2:**
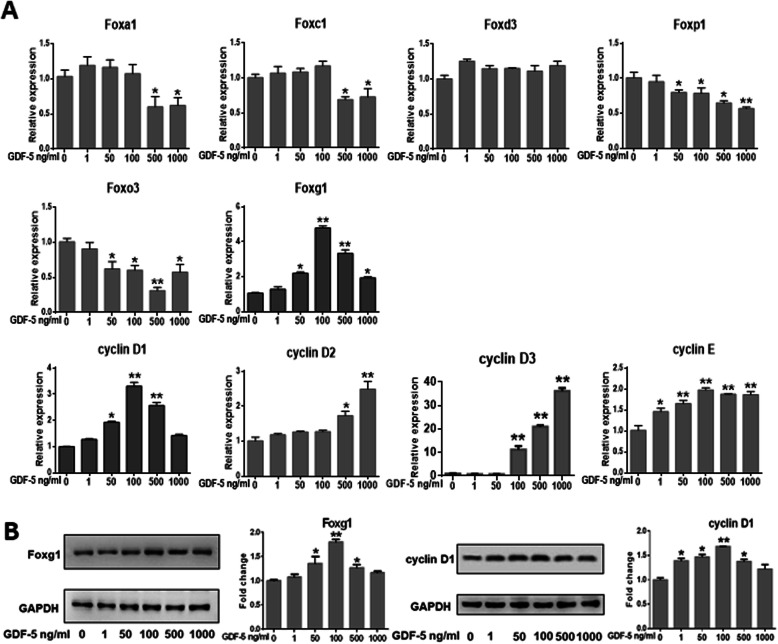
Changes of related factors after EpSCs were treated by GDF-5. Mouse primary EpSCs were treated with 0, 1, 50, 100, 500, and 1000 ng/ml of GDF-5 for 24 h. **a** The FOX family and cyclins genes were analyzed by qPCR. **b** The Foxg1 and cyclin D1 levels were analyzed by WB. The data are shown as the means ± SD of three independent experiments. *P < 0.05 vs. control (0 ng/ml GDF-5 as control), **P < 0.01 vs. control

In order to detect the possible downstream molecules of GDF-5, we consulted references and found FOX/cyclin may be the downstream molecules of GDF-5 [19]. Firstly, we screened downstream genes in the FOX and cyclins family, the results discovered that Foxg1 and cyclin D1 were a dose-dependent relationship with GDF-5. In addition, Foxg1 expression increased to 4.79-fold and cyclin D1 increased to 3.31-fold when the concentration of GDF-5 was 100 ng/ml (Fig. 2a). Secondly, the protein levels of Foxg1 and cyclin D1 in mouse EpSCs treated by GDF-5 were detected by WB. The results showed that there was a dose-dependent relationship between cells treated with GDF-5 and Foxg1/cyclin D1 protein expression. Moreover, Foxg1 increased to 1.79-fold and cyclin D1 increased to 1.68-fold when the optimal concentration of GDF-5 was 100 ng/ml (*P* < 0.01) (Fig. 2b).

### GDF-5 promotes EpSCs proliferation via Foxg1/cyclin D1 in vitro

**Fig. 3:**
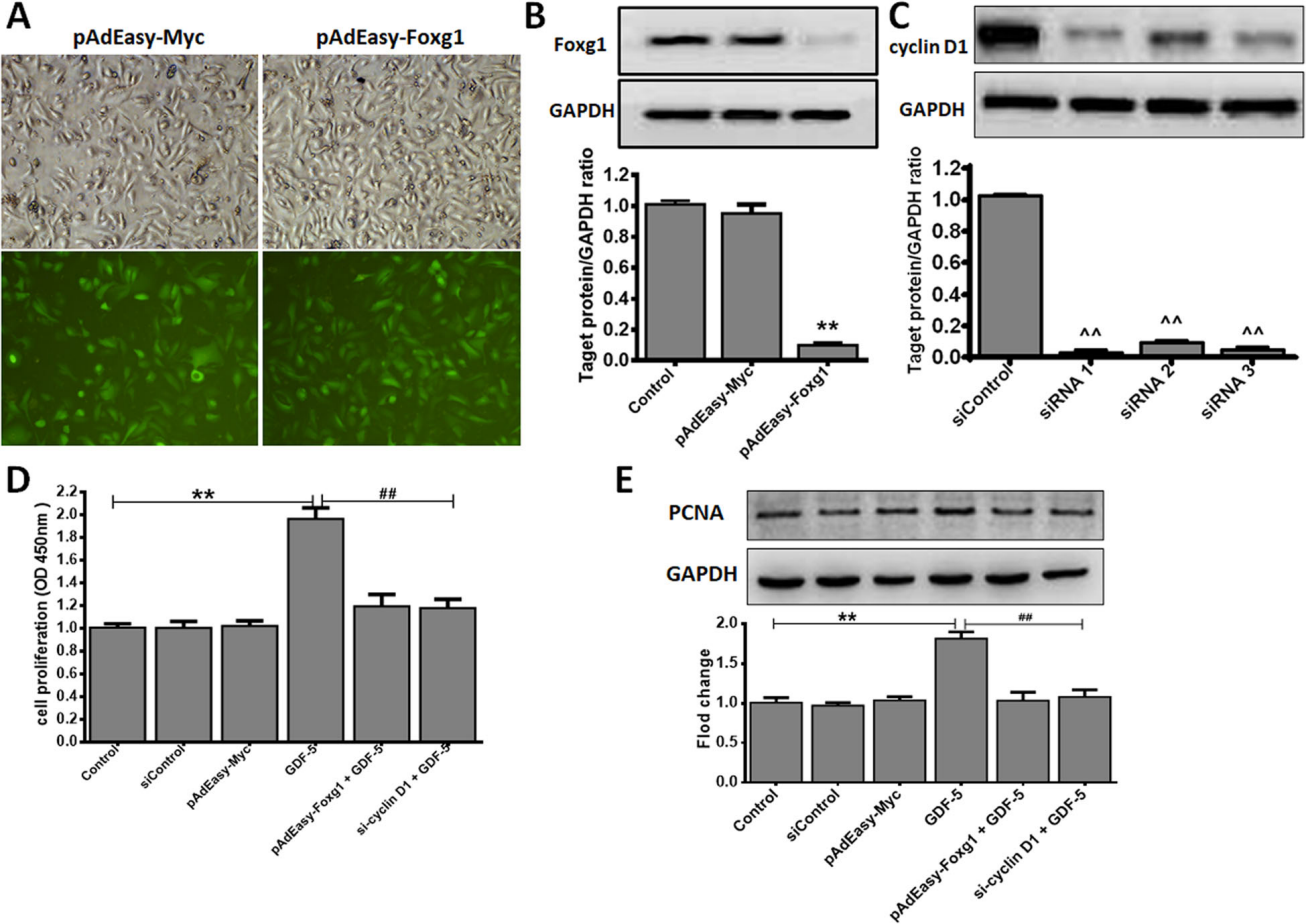
GDF-5 promotes EpSCs proliferation via Foxg1/cyclin D1 in vitro. Mouse EpSCs were infected with pAdEasy-Myc control adenovirus or pAdEasy-Foxg1. After the cells were transfected for 48 h with siRNA control plasmid or cyclin D1 siRNAs, mouse EpSCs were treated with 100 ng/ml GDF-5 for 24 h. **a** Fluorescence effects of pAdEasy-Myc and pAdEasy-Foxg1 infected mouse EpSCs (× 100 magnification). **b** Foxg1 protein expression level was analyzed by WB. Control: non-infected normal mouse EpSCs. **c** Three siRNAs were synthesized to inhibit cyclin D1 expression and quantification of WB. **d** Cell proliferation was measured by CCK-8. **e** The PCNA levels were analyzed by WB. The data are shown as the means ± SD of three independent experiments. **P < 0.01 vs. Control, *^^P* < 0.01 vs. siControl, ^*##*^*P* < 0.01 vs. the GDF-5 group

The above part proved that Foxg1 and cyclin D1 are the downstream molecules of GDF-5. Here we discuss the role of Foxg1 and cyclin D1 in GDF-5 promotes cell proliferation. We used adenovirus and siRNA infection technology to verify the interrelationship of Foxg1/cyclin D1 during cell proliferation. The pAdEasy-Myc transfection mouse EpSCs as control virus, the results showed that pAdEasy-Foxg1 and pAdEasy-Myc had a similar transfection efficiency, reaching to 90% and 95% (Fig. 3a), which indicated that each adenovirus successfully infected mouse EpSCs. The pAdEasy-Foxg1 group’s Foxg1 expression was reduced by 89% (*P* < 0.01) compared to the pAdEasy-Myc group, and there was no significance between the pAdEasy-Myc and the control group (Fig. 3b). Three siRNAs were synthesized to inhibit cyclin D1 gene expression. As shown in Fig. 3c, the siRNA1 (*P* < 0.01) with the best silencing efficiency was selected for subsequent research. Finally, mouse EpSCs were treated with 100 ng/ml GDF-5 or not treated for 24 h, and cell proliferation was evaluated by CCK-8 assay and PCNA protein analysis. Figure 3d and e showed that the GDF-5 group had a significant proliferation compared with other groups. In addition, the EpSCs proliferation was inhibited of pAdEasy-Foxg1 + GDF-5 group reduced 0.78 and cyclin D1 siRNA + GDF-5 group reduced 0.73 compared with GDF-5 group (Fig. 3e).

### The effect of GDF-5 on mouse EpSCs proliferation via Foxg1/cyclin D1 in vivo

**Fig. 4:**
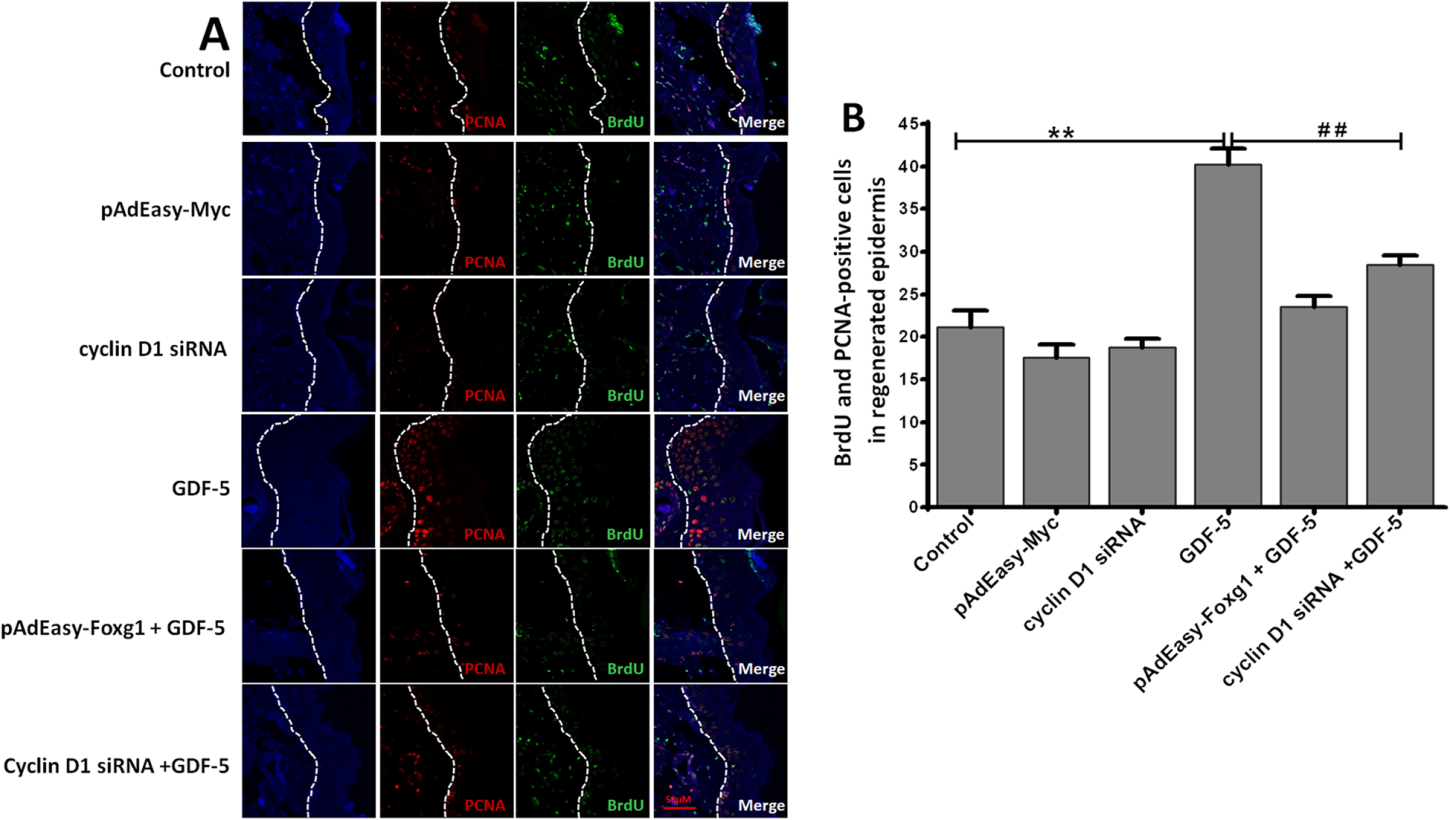
GDF-5 promotes EpSCs proliferation via Foxg1/cyclin D1 in vivo. The groups including control, pAdEasy-Myc, cyclin D1 siRNA, GDF-5, pAdEasy-Foxg1 + GDF-5, and cyclin D1 siRNA + GDF-5. **a** After mouse EpSCs were labeled by BrdU, BrdU^+^PCNA^+^-positive EpSCs were analyzed in wound. BrdU^+^ and PCNA^+^ cells in the regenerated epidermis are shown at the same magnification. Bar = 50 μm. **b** BrdU^+^ and PCNA^+^ cell count was performed using Image Pro Plus in the regenerated epidermis. The data are presented as the means ± SD of three independent experiments; **P < 0.01 vs. the control group, ^*##*^*P* < 0.01 vs. the GDF-5 group

To analyze the effect of GDF-5 on EpSCs proliferation in vivo, BrdU-labeled EpSCs and mouse model of burn injury were established as our previously described [29]. BrdU^+^ and PCNA^+^ EpSCs were presented by immunochemistry in the regenerated epidermis, and the double-positive EpSCs were counted in the different re-epithelialization area. As can be seen from (Fig. 4a, b), the number of double-positive cells increased to 40.18-fold in the GDF-5 group compared with the control group; the results showed that GDF-5 can promote EpSCs proliferation in vivo. However, the double-positive cells reduced 16.68 and 11.72 in the cyclin D1 siRNA + GDF-5 group and the pAdEasy-Foxg1 + GDF-5 group compared with the GDF-5 group, respectively. The data showed that the pAdEasy-Foxg1 and cyclin D1 siRNA abolished the effect of GDF-5 on the number of double-positive cells in the regenerated epidermis. Moreover, the double-positive cells increased in the pAdEasy-Foxg1 + GDF-5 group and the cyclin D1 + GDF-5 group compared with the control group, but the difference is not obvious.

### GDF-5 regulates cyclin D1 expression by Foxg1 and regulates transcriptional activity of the cyclin D1 gene promoter

**Fig. 5:**
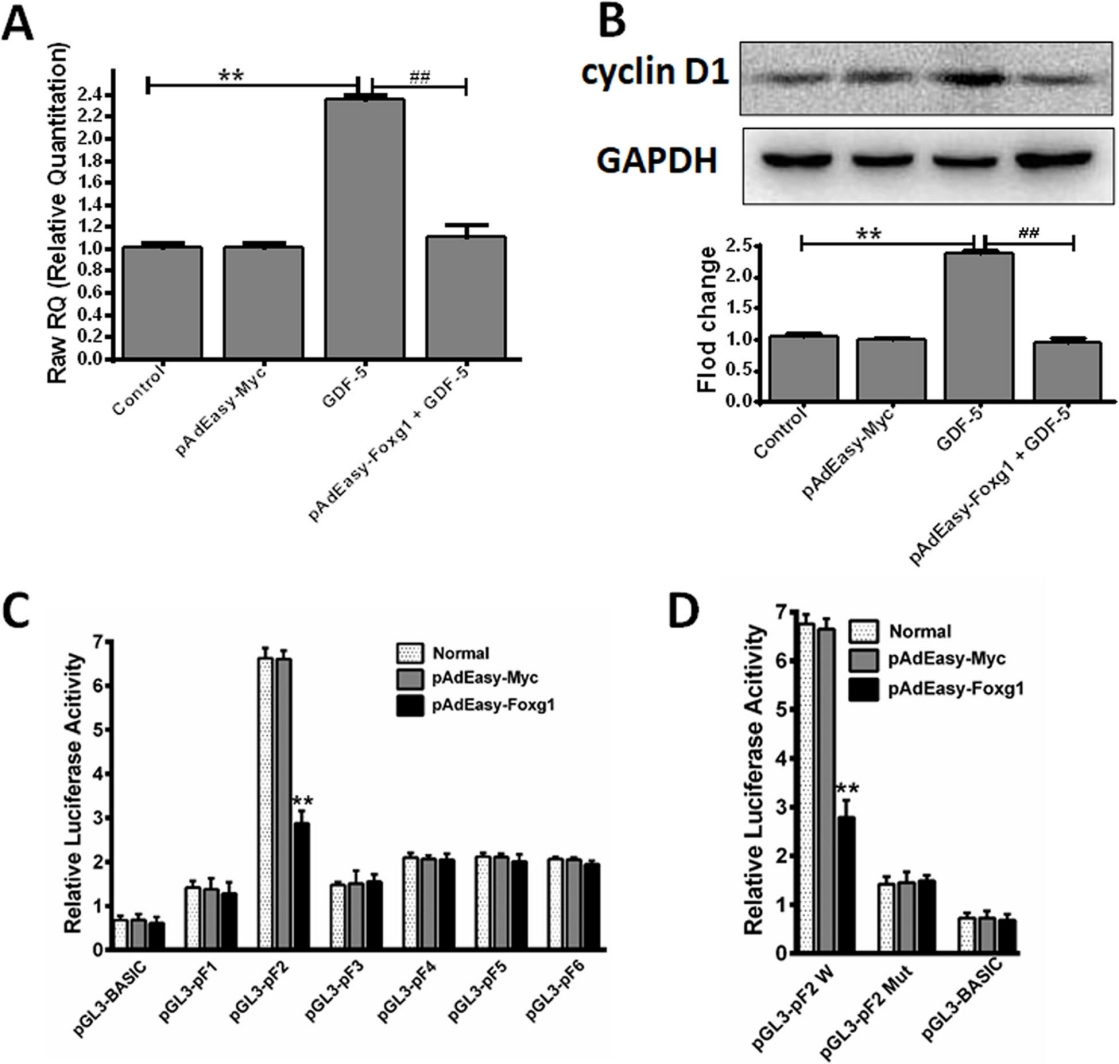
GDF-5 regulates cyclin D1 protein and mRNA expression through Foxg1. Mouse EpSCs were infected with pAdEasy-Myc control adenovirus or pAdEasy-Foxg1 and the cells were treated with 100 ng/ml GDF-5 for 24 h. **a** Cyclin D1 mRNA expression. **b** Cyclin D1 protein expression. **c**, **d** Mouse EpSCs were transfected with pAdEasy-Myc control adenovirus or pAdEasy-Foxg1 or the luciferase reporter expression vectors or mutated pGL3-pF2 vector using Lipofectamine 2000. The data are presented as the means ± SD of three independent experiments; **P < 0.01 vs. the control, ^*##*^*P* < 0.01 vs. the GDF-5 group

In order to analyze the transcription relationship between Foxg1 and cyclin D1, qPCR was used to detect cyclin D1 mRNA, and WB was used to detect cyclin D1 protein expression in the presence of pAdEasy-Foxg1. As can be seen from Fig. 5a, the expression of cyclinD1 mRNA in the GDF-5 group increased to 2.34-fold compared with the control group (*P* < 0.01), but the cyclin D1 mRNA expression of pAdEasy-Foxg1 + GDF-5 group was reduced back to the control level. At the same time, the cyclin D1 protein expression level also had the same trend (Fig. 5b). This showed that pAdEasy-Foxg1 blocked cyclin D1 expression. Next, we hypothesized that pAdEasy-Foxg1 inhibits cyclin D1 by exerting inhibitory activities to the cyclin D1 promoter. We constructed a pGL3-cyclin D1 (pF1, pF2, pF3, pF4, pF5, pF6) luciferase reporter gene expression vector. The dual-luciferase assay revealed that pAdEasy-Foxg1 significantly inhibited the activity of the cyclin D1 promoter (Fig. 5c). However, the mutant pGL3-pF2 did not respond to the pAdEasy-Foxg1 agonists (Fig. 5d).

## Discussion

At present, the effect of GDF-5 on wound healing has been reported [13, 15]. However, its specific mechanism for wound repairing is still unclear. In this paper, we discovered that GDF-5 promoted mouse EpSCs proliferation via the Foxg1/cyclin D1 signaling pathway in vivo and in vitro.

Other studies had reported that GDF-5 promote cell proliferation [13]; in this study, we found that GDF-5 can directly increase the number of EpSCs in vitro. We detected the effect of GDF-5 on EpSCs when the concentration of exogenous GDF-5 changed from 0 to 1000 ng/ml by CCK-8 assay at 12 h, 24 h, 48 h, and 72 h. The results showed that EpSCs had the best cell proliferation effect after being treated with 100 ng/ml exogenous GDF-5 (Fig. 1b), the effective concentration of GDF-5 on cells is similar to the previous reports [37]. In vivo, through the study of a deep partial-thickness burn mouse model, we found that GDF-5 promoted the proliferation of EpSCs, which is consistent with the results of in vitro experiments (Fig. 4a, b). In addition, PCNA is a marker that reflects the state of cell proliferation [36]; we tested the expression of PCNA protein after different concentrations of GDF-5 treatment for 24 h (Fig. 1c). Combining the results of cell count and PCNA protein analysis, it was determined that GDF-5 promoted EpSCs proliferation in vitro and in vivo.

**Fig. 6:**
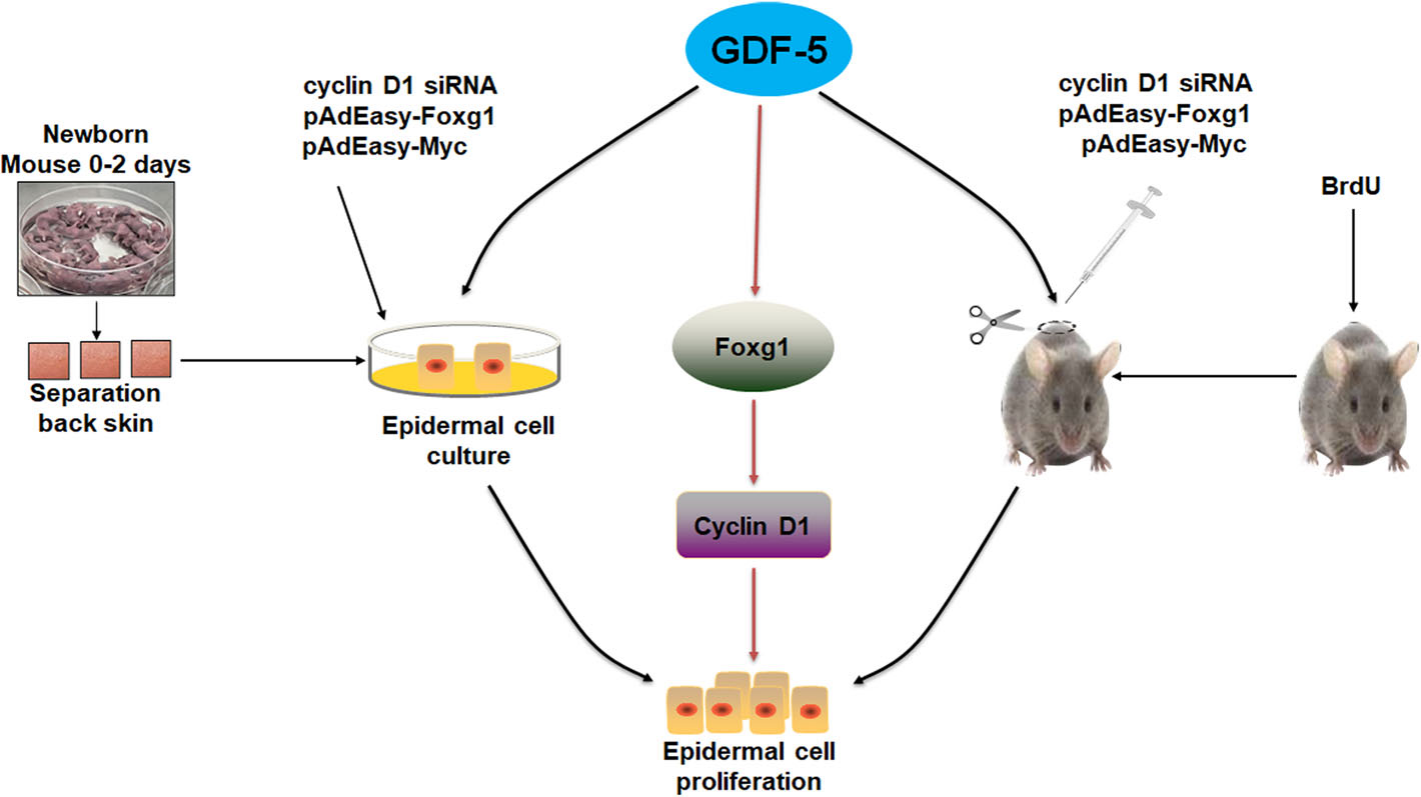
GDF-5 promotes epidermal stem cell proliferation via Foxg1-cyclin D1 signaling

FOX and cyclin have important functions in the proliferation of many cell types [21, 38]. Firstly, we screened several subfamilies of the FOX family and cyclins related to cell proliferation by qPCR. Here, it was found that Foxg1 and cyclin D1 increased significantly (Fig. 2a). Wang Fan et al. found that cyclin D1 was significantly expressed during the proliferation of human EpSCs [29]. We previously reported that nitric oxide induces FoxG1 expression in human EpSCs [32]. In the analysis on GDF-5 promoting EpSCs proliferation, we found that Foxg1 and cyclin D1 could have prevented the proliferation effect of GDF-5 (Figs. 3c, d and 4); further analysis from the protein level found that Foxg1 and cyclin D1 were positively regulated by GDF-5 (Figs. 2b and 3e). Federica Verginelli et al. found that a transcriptional program regulated by Foxg1 is significant for promoting glioblastoma growth [39]. Combined with the results of in vivo and in vitro studies, this indicated that after GDF-5 stimulates EpSCs; the downstream molecules Foxg1 and cyclin D1 were activated (Fig. 6).

Besides, Foxg1 is involved in inhibiting the cell cycle exit initiated by p21 [27]. Cyclin D1 is a key regulator of cell proliferation by promoting cell cycle transition, and its expression is regulated by transcription level [40, 41]. To clarify the upstream and downstream relationship between Foxg1 and cyclin D1, dual-luciferase reporter gene analysis was used, and we found that GDF-5 induced cyclin D1 transcription was regulated by Foxg1-mediated cyclin D1 promoter activity (Fig. 5c, d). There may be other signaling pathways for GDF-5 to promote EpSCs proliferation. From Fig. 2a, discovered Foxo3/Foxp1 decreased significantly and cyclin D2/cyclin D3 increased significantly at the transcription level. Foxo3 and Foxp1 have been reported to play an inhibitory role in cell proliferation [19], and cyclin D2/cyclin D3 helps isolate cell transplant factor p27 [42]. Whether GDF-5 regulates these genes to promote the proliferation of EpSCs will be analyzed in another project.

## Conclusions

This study shows that GDF-5 plays an important role in EpSCs proliferation in vitro and in vivo. The proliferation is regulated by activating Foxg1-cyclin D1 signaling pathway. The results can initially determine that GDF-5 can be used as a new target for wound repairing.

## Data Availability

The data that support the findings of this study are available from the corresponding author upon reasonable request.

## Abbreviations

EpSCs: Epidermal stem cells
GDF-5: Growth/differentiation factor 5
MAPK: Mitogen-activated protein kinase
PCNA: Proliferating Cell Nuclear Antigen
Wnt: Wingless/Integrated
BMP: Bone morphogenetic protein
FOX: Fork head box
TGF-β: Transforming growth factor-beta
CDK: (4 or 6) Cyclin-dependent kinase 4 or 6
BPE: Bovine pituitary extract
CCK-8: Cell counting kit-8
qPCR: Real-time quantitative PCR
WB: Western blotting
FC: Flow cytometry
PI3K-AKT/PKB: Phosphoinositide-3-kinase–protein kinase B/Akt
